# Evaluation of the Wound Healing Activity of the Crude Extract of Leaves of* Acanthus polystachyus* Delile (Acanthaceae)

**DOI:** 10.1155/2018/2047896

**Published:** 2018-06-11

**Authors:** Wubante Demilew, Getnet Mequanint Adinew, Seyfe Asrade

**Affiliations:** ^1^Department of Pharmacy, Bahir Dar Health Science College, Bahir Dar, Ethiopia; ^2^Department of Pharmacology, School of Pharmacy, College of Medicine and Health Sciences, University of Gondar, Gondar, Ethiopia

## Abstract

**Background:**

Medicinal plants play indispensable roles to treat various ailments.* Acanthus polystachyus* is one of the medicinal plants used traditionally for treatment of wounds. However, there were no scientific reports documented so far on the wound healing activities of this plant. Thus, the present study provides a scientific evaluation for the wound healing potential of the crude extract of* A. polystachyus* leaves.

**Methods:**

The crude extraction was carried out using 80% methanol. The crude extract was prepared in 5% (w/w) and 10% (w/w) ointment and evaluated for wound healing activity using excision, infected, and incision wound models in Swiss albino mice.

**Results:**

Both 5%  and 10% (w/w) ointments significantly reduced period of epithelialization and increased wound contraction rate and tensile strength compared to the negative control group (*P* < 0.05). The wound healing activity of 10% (w/w) ointment treated group was greater than 5% (w/w) and nitrofurazone ointment treated groups in* S. aureus* infected wound model.

**Conclusion:**

These results demonstrate that the crude extract of* A. polystachyus* leaves possesses wound healing activities. This justifies the traditional claimed use of the plant for treating uninfected and infected wounds caused by* S. aureus*.

## 1. Background

Wound defined as the cellular and anatomic disruption of a tissue may be caused by chemical, physical, microbial, thermal, or immunological damage to the tissue [1]. Wound healing is restoration of structure and function of an injured tissue in order to approximate prewound characteristics. The effective management of wounds will reduce the number of complications and allow rapid return to normal function [2]. About 70% to 90% of populations in some industrialized nations and between 70% and 95% of citizens in the majority of developing countries use traditional medicine as primary healthcare to address their healthcare needs and concerns [3]. Several medicinal plants were scientifically proven to be used for the treatment of wounds and some other medicinal plants are mentioned in the Ethiopian folk medicine for wound management [4].

*Acanthus* is a genus of flowering plants belonging to the family Acanthaceae. The* Acanthus* family is fairly large with some 2500-3000 species in about 250 genera. The Acanthaceae family possesses antifungal, cytotoxic, anti-inflammatory, antipyretic, antioxidant, antiviral, insecticidal, hepatoprotective, immunomodulatory, and antiplatelet activities [5, 6].

*Acanthus polystachyus *Delile is a shrub or small tree (to 7 meters) that is ecologically widespread from medium to high altitudes (1000-3200 m) in different parts of Ethiopia. This plant commonly grows in the vicinity of Blue Nile River, around Bahir Dar city [7]. This species has pink flowers and soft, hairy leaves.* A. polystachyus *is native to Burundi, Rwanda, Uganda, Sudan, Ethiopia, Kenya, and Tanzania.* A. pubescens *(Oliv.) Engl. is used in traditional medicine for treatment of syphilis and gonorrhea in Tanzania [8].

The decoction of the leaves is used for the treatment of gastroenteritis, pneumonia, and anthrax. It is also reported that a preparation of the dried leaves is used externally as a remedy for scabies in Rwanda [8].* A. polystachyus* is traditionally used for treating scorpion sting with root decoction given orally and to treat bleeding and stabbing pain leaf paste is applied. The leaves are used as a medicine with butter and applied to wounds in Ethiopia [9].

In previous studies, 80% ethanol extract of the leaves exhibited antifungal activity, weak antibacterial activity, and antiviral activity against polio and measles viruses [10].

The purpose of this study was to evaluate the wound healing activities of the crude extracts of leaves of* A. polystachyus *Delile, which can in turn provide a basis for isolation and identification of pharmacologically active compounds.

## 2. Methods

### 2.1. Collection, Authentication, and Preparation of Plant Material

Fresh leaves of* A. polystachyus *Delile were collected in February 2017 from the border of Blue Nile River (around Bahir Dar city, Amhara Regional State). The specimen was authenticated and deposited in Department of Biology, College of Natural and Computational Sciences, University of Gondar, for future reference with a voucher specimen number of DW 001. The leaves were washed and allowed to dry under the shade. The dry leaves were ground to coarse powder using mortar and pestle.

### 2.2. Methods of Extraction

The leaves of* A. polystachyus* were ground into a coarse size using mortar and pestle. Then, the coarse powder was subjected to crude extraction by maceration protocol. Briefly, 150 g of powdered material was weighed and soaked in a flask containing 500 ml of 80% methanol in water (with a total of 1500 g powder in 5 L solvent) for a period of 3 days with occasional shaking using a shaker at room temperature. The extract was filtered using Whatman No. 1 filter paper. The residue was remacerated for the second and third times with fresh solvent for a total of 6 days in order to obtain a better yield.

After filtration, the three extracted solutions were combined and concentrated using a hot air oven with a temperature not exceeding 40°C to remove the solvents. Then, the concentrated filtrate was dried by using desiccators to remove its aqueous content. Finally, the dried extract was packed in a closed vessel and stored in deep freezer until being required for the experiment.

### 2.3. Ointment Formulation

Simple ointment B.P. was prepared using hard paraffin, cetostearyl alcohol, white soft paraffin, and wool fat. The master formula used for the preparation of ointment was taken from British Pharmacopoeia [11].

**Table:** 

**Ingredients **	**M.F**	**R.F**
Wool fat…………………………………	50 g	10g
Hard paraffin……………………………	50 g	10g
White soft paraffin………………………	850g	170g
Cetostearyl alcohol………………………	50g	10g
	**1000g**	**200g**

M.F is Master Formula; R.F is Reduced Formula. The 200 g of simple ointment base was prepared by placing hard paraffin (10 g) in a beaker and melted over water bath. The other ingredients such as cetostearyl alcohol (10 g), white soft paraffin (170 g), and wool fat (10 g) were added in descending order of melting point, respectively, after removing from melting.

All the ingredients were melted over a water bath with constant stirring until they became homogeneous. The mixture was removed from the heat and stirred until cold. To prepare hydroalcoholic extract ointment, 10 g and 20 g of the powdered extract were incorporated into portion of simple ointment base to prepare 5% and 10% (w/w) ointment, respectively, by levigation. The remainder of simple ointment base was gradually added and mixed thoroughly. Finally, the extract ointment was transferred to a clean container for topical application during the experiment [12].

### 2.4. Phytochemical Screening

Photochemical screening test was performed on the crude extract and solvent fractions following standard procedures [13–15].

### 2.5. Acute Dermal Toxicity

The test was performed according to the OECD draft guideline number 434 [16]. For acute dermal toxicity, a total of ten female Wistar rats aged between 8 and 12 weeks were used. They were divided into two groups of five animals each for treatment and control groups. Animals with normal skin texture were housed individually in a cage and acclimatized to the laboratory condition for five days prior to the test. Following acclimation, around 10% of the body surface area fur was shaved 24 h before the study from the dorsal area of the trunk of the test animals.

First, a sighting study was performed to determine the starting dose by applying 2000 mg/kg of the 10% extract ointment. There was no death or skin irritation observed within 24 h, and then four additional rats from each group were used, and the same dose of the extract ointment was applied. That is, a limit test dose of 2000 mg/kg of the 10% ointment formulation of the extract and control groups were applied uniformly over the shaved area and observed for 24 h. At the end of the exposure period, residual test substance was removed and the animals were observed for development of any adverse skin reactions daily for 14 days [17].

### 2.6. Wound Healing Activity Testing

#### 2.6.1. Grouping and Dosing of Animals

Healthy, adult white albino mice of either sex (25–35 g, and 6–8 weeks of age) were used. Four groups of mice, each containing six mice, were used for excision model. Animals in group I were treated with simple ointment (as negative control), whereas group II was treated with nitrofurazone (0.2 %) ointment (as a standard drug). Group III and group IV were treated with 5% (w/w) and 10% (w/w) extract ointments, respectively. Four groups of mice, each containing six mice, were used for incision wound model. The animals of groups I-IV were treated in a similar fashion with excision wound model and infected wound model. All the experiments were conducted in accordance with the internationally accepted guideline for laboratory animal use and care [18].

#### 2.6.2. Excision Wound Model

On wounding day, animals were anesthetized using subcutaneous injection of ketamine (1 ml/kg) and diazepam (1 ml/kg). After wound area preparation with 70% alcohol, the dorsal fur of the animals was shaved with shaving machine and the anticipated area of the wound to be created was outlined on the back of the animals on the dorsal thoracic region 1 cm away from vertebral column on the anesthetized mouse. Full thickness circular excision wounds sized about 300 mm^2^ were created along the markings using toothed forceps, scalpel, and scissors. Hemostasis was achieved by blotting the wound with cotton swab soaked in normal saline. The entire wound was left open.

The mice were divided into four groups (6 mice per group) randomly and each mouse was placed in a separated cage. The treatment was done once daily topically in all the cases. The wounding day was considered as day 0. The standard drug, extract, and simple ointment were applied topically to the respective groups till the wound was completely healed [19–21].

#### 2.6.3. Infected Wound Model

Full thickness circular excision wounds sized about 300 mm^2^ were created along the markings using toothed forceps, scalpel, and scissors using similar procedures to those in the excision wound model. After achieving complete haemostasis by blotting the wound with cotton swab soaked in normal saline, the wound was inoculated with standard strain of* S. aureus *culture (24 hrs) obtained from Department of Microbiology, School of Medicine and Health Sciences, University of Gondar. After 24 h of contamination with* S. aureus* suspensions (1×10^6^ CFU/mL), the experimental animals were randomized into four groups (1-4). The animals were placed singly in separate cages and treated in similar procedures to those followed in the case of excision wound model. Treatments of infected wounds commenced on the 2nd day to allow establishment of infection on the wound. The wound area was measured with a translucent paper and a 1 mm^2^ graph sheet on day 0, day 2, day 4, and thereafter every other day until wound closure is completed was recorded. Wound contraction was calculated as a percentage of the original wound size [22]. Throughout the experiment, presence or absences of phlogistic characteristics (infiltration, edema/localized swelling, abscess, or lesion and exudates) were monitored every 24 hours [23].

#### 2.6.4. Measurement of Wound Contraction

The wound closure rate was assessed by tracing the wound on days 2, 4, 6, 8, 10, 12, 14, and 16 using transparent paper and a permanent marker. In case of infected wound model, measurement of wound contraction was prolonged till 18 days. The wound areas recorded were measured using 1 mm^2^ scale of graph paper. Changes in wound area were evaluated, giving an indication of the rate of wound contraction and epithelialization period. The evaluated surface area was used to calculate the percentage of wound contraction, taking initial size of the wound as 100% [20] as shown below:

(1) %  Wound  closure=wound  area  on  1st  day−Wound  area  on  day  nWound  area  on  1st  day×100

where *n* is number of days (2nd, 4th, etc.).

#### 2.6.5. Epithelialization Period Measurement

Falling of scab leaving no raw wound behind was taken as end point of complete epithelialization and the days required for this were taken as period of epithelialization [20].

#### 2.6.6. Incision Wound Model

Animals were anesthetized in the same manner described for excision wound model. The dorsal fur of each mouse was then shaved and a 3 cm long longitudinal paravertebral incision 1 cm away from vertebral column was made through the skin and subcutaneous tissue. The parted skin was then sutured 1 cm apart using a surgical thread (silk no. 00 round) as described by Ehrlich and Hunt with slight modification [24]. After 24 h of wound creation (on 1st day), animals were treated as described under grouping section, with topical formulation of nonmedicated simple ointment, extract, and standard drug once daily for nine days. The suture was removed on day 8 after incision and tensile strength was measured on the 10th day after wounding using continuous water flow technique [25, 26].

#### 2.6.7. Measurement of Tensile Strength

**Figure 1:**
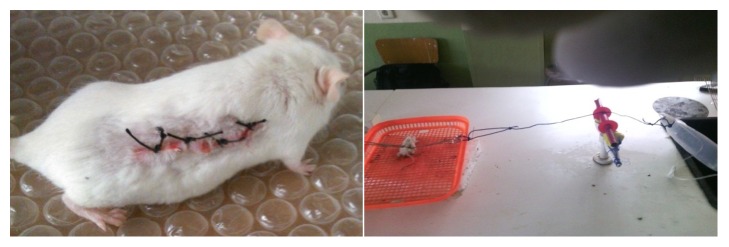
Photograph of incision wound on day 0 and measurement of tensile strength on 10th day using water flow technique.

Tensile strength (the force required to open the healing skin) was used to measure the extent of wound healing. The model used for this purpose consists of fixed shelves with a table. There are two Allis forceps, one is fixed to the opposite side of shelve and another is tied with rope that was attached to the empty IV bag on which the weights are placed. On the 10th day after wounding, each mouse was anesthetized using diethyl ether to secure animal to the table. The two forceps were firmly applied 1 cm away from healed tissue on the incised part of the skin onto the line facing each other. Water is allowed to flow into bag from tap water through IV line. A gradual increase in weight was transmitted to the wound site pulling apart the wound edges. As soon as wound gaping appeared, water flow was stopped, and the volume of water collected in the container was determined and noted as an indirect measure of breaking strength in grams. Percentage of tensile strength for extract and reference drug with respect to negative control treated with simple ointment (SO) was measured using the following formula [25] (Figure 1):

(2) Tensile  strengthTSof  extractstandard  %=TS  extract/standard – TS  S.OTS  S.O  x  100

### 2.7. Data Management, Processing, and Analysis

The data was entered, coded, and analyzed using Statistical Package for the Social Sciences (SPSS) version 20. The experimental data was expressed as mean ± Standard Error of the Mean (SEM). Finally, one-way analysis of variance (ANOVA) followed by post hoc Tukey's test was employed and* P* value < 0.05 was considered statistically significant.

## 3. Results

The percentage yield value of the crude extract was 14%. In acute dermal toxicity test, the application of 10% w/w with limit dose of 2000 mg/kg did not show any sign of inflammation and edema. There was also neither mortality nor any sign of toxicity observed in rats when monitored for 14 days after topical application of the extract.

### 3.1. Phytochemical Constituents of the Crude Extract and Solvent Fractions

According to the qualitative phytochemical screening study, the crude extract of the leaf of* A. polystachyus* was found to be positive for the presence of tannins, flavonoids, saponins, polyphenols, terpenoids, glycosides, and anthraquinones, whereas alkaloids and steroids were absent.

### 3.2. Excision Model

#### 3.2.1. Wound Contraction

**Figure 2:**
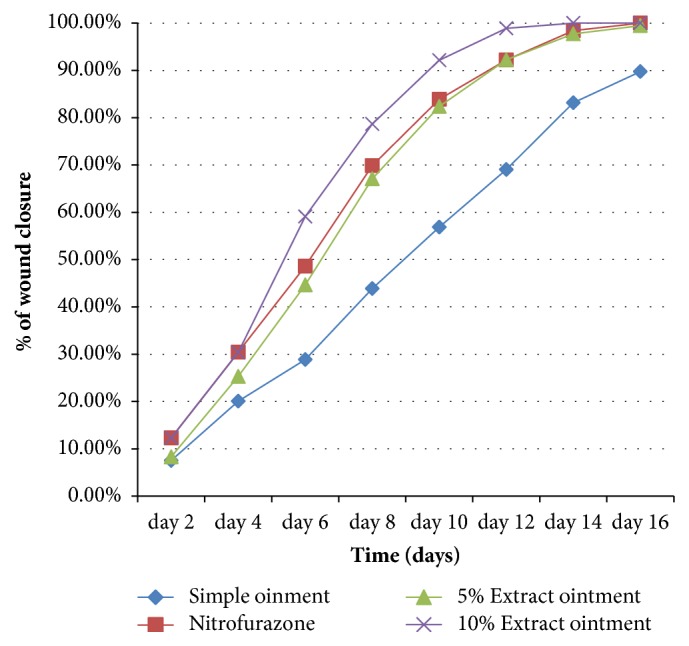
Effects of the 80% methanolic extract of* A. polystachyus* D. leaves on the percentage of wound closure of excision wound model in mice.

Topical applications of ointments of the 80% methanolic extracts of* A. polystachyus* leaves showed significant effect on wound healing process in mice. The progress of wound contraction induced by treatment of 5% (w/w) and 10% (w/w) ointment of 80% methanolic extract, simple ointment base, and nitrofurazone 0.2% (w/w) ointment is shown in Table 1. The plant extracts facilitated wound contraction significantly at both dose levels from 6th day to 16th day as compared to negative control. The 10 % (w/w) crude extract ointment treated group showed significant (*P* < 0.05) wound contraction starting from day 6. This effect was highly significant (*P* < 0.001) from 6th day onward in comparison with the control group (simple ointment). There was no significant difference in wound healing activity between the 10% (w/w) and 5% (w/w) extracts, but higher rate of wound closure was observed with 10% (w/w) ointment. The maximum percentages (rate) of wound contraction were observed in animals treated with 10% extract ointment from the 10th to 14th day, which were 92.1 % and 100 %, respectively. Similar percentages of wound contraction (92.2%  and 100%) were observed in animals treated with the standard drug from the 12th to 16th day. Hence, the 10% extract ointment revealed better observable effect compared to the standard drug; however, it failed to reach statistical significance (Figure 2).

The animals treated with 5% (w/w) methanolic extract ointment showed significant wound contraction from 6th day onward as compared to control group (*P* < 0.05). Significant wound contraction was also observed for nitrofurazone 0.2% (w/w) ointment treated group from 6th day onward as compared to control group (*P* < 0.01). The maximum percentages of wound contraction for nitrofurazone 0.2% (w/w) ointment were seen in the 12th, 14th, and 16th days, which were 92.2, 98.4, and 100%, respectively. However, there was no significant difference in wound healing activity between 5% and 10% extracts and the standard drug except 5% and 10% extracts at days 6 and 10. Furthermore, complete wound closure was observed in 10% (w/w) extract and standard ointment treated groups within 14 and 16 days, respectively (Table 1).

#### 3.2.2. Epithelialization Period

The time for complete epithelialization was short in extract ointment and nitrofurazone treated groups as compared to control (simple ointment treated group). On average, the period of epithelialization was 20.9, 15.3, 15.8, and 13.2 % for control group, standard drug, and 5% (w/w) and 10% (w/w) extract ointment, respectively. The 10% extract ointment treated group showed faster rate of epithelialization (*P* < 0.001) compared to control group. Similarly, 10% (w/w) extract ointment showed significant (*P* < 0.05) difference of epithelialization period as compared to 5% (w/w) extract ointment treated group. Moreover, the 10% (w/w) extract showed higher percentage of decrease in epithelialization periods than nitrofurazone but failed to reach statistical significance.

Animals treated with 10% (w/w) extract, 5% (w/w) extract, and nitrofurazone ointments showed 36.8%, 24%, and 26.4% decrease in epithelialization period as compared to control group, respectively (*P* < 0.001 for all cases). However, 10% (w/w) extract has slightly higher percentage of decrease in epithelialization period as compared to standard drug but failed to reach statistical significance (Table 2).

### 3.3. Infected Wound Model

#### 3.3.1. Wound Contraction

**Figure 3:**
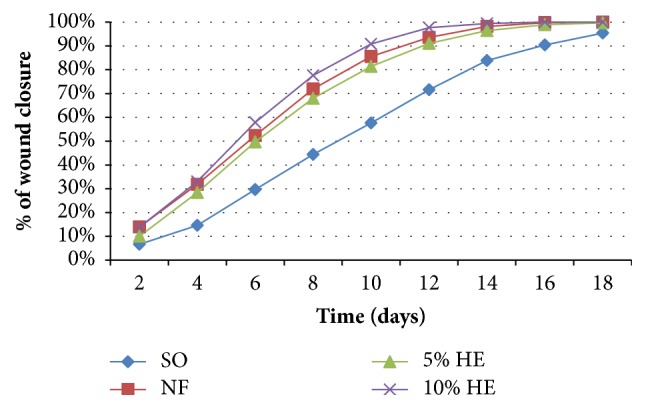
Effects of 80% methanolic crude extract ointment of* A*.* polystachyus *leaves on percentage of wound closure of infected wound model in mice.

In this experiment, before treatment, the wounds in all animals exhibited phlogistic characteristics (infiltration, blister formation, edema, and exudates). These characteristics vanished in groups treated with 10% extract ointment, 5% extract ointment, and nitrofurazone ointment within 4 to 6 days of treatment. However, the group treated with simple ointment exhibited these phlogistic characteristics for one weak and more. After follow-up period, the negative controls were treated with nitrofurazone ointment but three of the mice passed away on the 19th and 21st days possibly due to systemic infections. The ointments of crude extract revealed significant effect of wound healing in mice infected with* S. aureus.* The rate of wound healing was faster for the groups treated with 10% ointment as compared to nitrofurazone and 5% ointment. Wound area contraction was promoted and completely healed within 16 and 18 days for group treated with 10% extract followed by standard drug (nitrofurazone) and 5% extract ointment treated groups. The 10% (w/w) extract ointment treated group showed significant wound contraction starting from day 4 (*P* < 0.05) and from 6th day onward in comparison with the negative control group (*P* < 0.001). The maximum percentages of wound contraction were seen from days 12, 14, and 16, which were 97.7, 99.4, and 100%, respectively. Likewise, animals in groups treated with 5% (w/w) extract ointment and nitrofurazone showed significant (*P* < 0.05) wound contraction from 4th day onward as compared to negative control group. The maximum rates of wound closure for nitrofurazone 0.2% (w/w) ointment were seen on the 12th, 14th, 16th, and 18th days, which were 93.5, 98.2, 99.97, and 100%, respectively (Figure 3). Even though higher rate of wound closure was observed with 10% (w/w) ointment, there was no statistically significant difference between the 10% and 5% extracts and the standard drug (Table 3).

#### 3.3.2. Epithelialization Period

The period of epithelialization was short in extract ointment and nitrofurazone treated groups as compared to negative control. The epithelialization period was reduced in a dose-related manner from 17.00 ± 0.86 for the 5% ointment to 14.17 ± 1.17 for the 10% ointment treated groups. On average, the period of epithelialization was 16.2, 17.0, and 14.2 for standard drug, 5% (w/w) extract ointment, and 10% (w/w) extract ointment, respectively.

The 10% extract ointment and 5% extract ointment reduce the period of epithelialization in a dose-dependent manner (17.00 and 14.2, resp.). The 10% extract ointment treated group showed faster rate of epithelialization (*P* < 0.001) compared to the negative control group. Moreover, 10% (w/w) extract ointment showed significant (*P* < 0.05) difference of epithelialization period as compared to 5% (w/w) extract ointment treated group. However, the observable difference between epithelialization periods of the extract ointment (5% and 10%) treated groups and standard drug treated group failed to reach statistical significance. Groups treated with simple ointment failed to reepithelize within the follow-up periods (Table 4).

### 3.4. Incision Model

#### 3.4.1. Tensile Strength

The 10% (w/w) extract, standard drug, and 5% (w/w) extract treated groups showed significant increase in breaking strength by 35.8, 32.7, and 31.2%, respectively, when compared to the negative control (*P* < 0.001). In this finding, the increase in tensile strength was found to be higher in 10% extract ointment as compared to nitrofurazone and 5% (w/w) extract treated groups but failed to reach statistical significance (Table 5).

## 4. Discussion

Traditionally, the leaves of* A. polystachyus* are used for wound healing activity mixed with butter, which is hydrophobic. Applying the extract directly on the affected wound cannot bring the desired effect as it does not stay longer on the wounded skin of the experimental animals. Ointment is necessary to achieve a sustained drug release at the application sites. Hence, a hydrophobic base was selected based on traditional claim and active metabolites of leaves of* A. polystachyus* predominate polar components, which would be released better from the nonpolar base and vice versa [27]. The ointment base has additional roles like formation of occlusive barrier for moisture by hard and white soft paraffin. Wool fat and cetostearyl alcohol are thickeners and they are used for stabilization of ointment [12].

The results of this study on wound healing activity revealed that the crude extract significantly increases wound healing effects with both 10% (w/w) and 5% (w/w) extract ointment treated groups in the excision, infected, and incision wound models. This can be supported by the fact that the greater the reduction in the rate of wound contraction is, the better the efficacy of medication is and the wound will close at faster rate if the medication is more efficient [28].

In excision wound healing model, the crude extract (80% methanol) of the leaves of* A. polystachyus* showed statistically significant wound area contraction compared to the negative control. The 10% (w/w) extract ointment treated group revealed faster wound area contraction from day 6 to day 14, whereas the 5% (w/w) extract ointment treated group showed statistically significant wound area contraction starting from the 8th day onwards. The higher wound contraction rate of the extract ointment may be due to either its dose-dependent antibacterial effect or induction of macrophage cell proliferation [29].

Furthermore, the period of epithelialization was significantly reduced from 20 days (negative control) to 15, 15, and 13 days for 5% extract, nitrofurazone, and 10% extract ointment treated groups, respectively. The shorter period of epithelialization and faster wound area contraction could be due to the ability of* A. polystachyus* leaf extract to enhance collagen synthesis, induction of cell proliferation, and antimicrobial activities of bioactive constituents [30].

In the case of infected wound model, the ointments of crude extract revealed statistically significant wound healing effect in mice infected with* S. aureus*. The infiltration, blister formation, edema, and exudates exhibited on the wounds of mice before treatment vanished in all treated groups except the negative control. Groups treated with 10% extract ointment showed faster rate of wound contraction than nitrofurazone and 5% extract ointment treated groups. Additionally, the period of epithelialization was shorter in 10% extract followed by nitrofurazone and 5% extracts. This finding indicated that the wound healing activity of the extract in infected wound model was presumed to be dose-dependent. In this study, the antibacterial activity of the extract was confirmed against common wound infecting pathogens, which might contribute remarkably to the faster wound healing rate. Supporting evidence explained that the eradication of the colonizing organisms from infected wounds creates a suitable environment for wound healing to take place. As a result, the antimicrobial activity reported in infected wound model shows the promising potential of* A. polystachyus* towards wound management [22, 23]. This is further strengthened by the fact that chloroform fractions of* A. ilicifolius* leaves have in vitro antibacterial activity against common skin infection pathogens such as methicillin-resistant* Staphylococcus aureus*,* Streptococcus pyogenes, Pseudomonas aeruginosa, Candida albicans*, and* Trichophyton rubrum *which could support the wound healing activities [31]. Moreover, studies revealed that medicinal plants such as* Dissotis theifolia* [22] and* Piper hayneanum* [23] which have antibacterial and antifungal activities also possess wound healing effects.

In incision wound model, significant increase in skin breaking strength was observed. Groups treated with 10% and 5% (w/w) extracts and standard ointments showed statistically significant increase in tensile strength as compared to simple ointment base treated group. However, the difference in tensile strength was not statistically significant among standard drug and 10%  and 5% (w/w) ointment treated groups. The increase in tensile strength in the incision model may be due to the antioxidant activity of the extract, increase in collagen synthesis and maturation, formation of stable intra- and intermolecular cross-link, matrix deposition, and cell migration. For instance, iridoid glycosides isolated from the Acanthaceae family and flavonoids are known in promoting wound healing via inhibition of collagen synthesis [5, 28, 32].

Another possible reason for enhanced wound healing effect could be due to the crude extracts of* A. polystachyus* leaves which may possess antioxidant, free radical scavenging properties and promote cell proliferating properties. The role of antioxidant and free radical scavenging property in wound healing process is further strengthened by other studies conducted on the Acanthaceae family, which revealed that the plant possesses anti-inflammatory, antipyretic, and antioxidant properties [5]. To mention some, a study on the leaf and root extracts of* A. ilicifolius* showed antioxidant activity and scavenging free radicals (superoxide and hydroxyl radicals), due to the presence of flavonoids [33]. In addition, the root extract of* Acanthus sennii* revealed the presence of glycosides, flavonoids, and polyphenols. in particular, iridoid glycosides isolated and validated in the Acanthaceae family possess antioxidant, antimicrobial, analgesic, antitumor, and anti-inflammatory properties [5, 34].

The role of phytochemicals in wound healing is also supported by different studies. For instance, tannins are seen to be active detoxifying agents and inhibit bacterial growth [35]; terpenoids promote the wound healing process mainly due to their astringent and antimicrobial property [36]; flavonoids are potent antioxidants, free radical scavengers [28, 32]. Polyphenols and flavonoids (prevent the synthesis of prostaglandins) possess anti-inflammatory properties and have antimicrobial activities [37]. Glycosides (iridoid glycosides) isolated from the same family (Acanthaceae) possess antioxidant, antimicrobial, analgesic, antitumor, immunomodulatory, and anti-inflammatory effects [34]. Therefore, the presence of phytochemicals in the crude extract such as terpenoids, flavonoids, glycosides, saponins, tannins, and phenolic compounds may contribute to wound healing activities independently or synergistic effects.

## 5. Conclusion

In this study, in all the three models, the different phases of wound repair, wound contraction, epithelialization, and tensile strength, were enhanced by the 80% methanolic crude extract ointment of the leaves of* A. polystachyus* as compared to the negative control group. These results collectively demonstrate that the 80% methanolic extract possesses wound healing activity and this justifies the use of the leaves of* A. polystachyus* for treatment of wounds as claimed in the folklore literature. This study also showed that the crude extract of* A. polystachyus* was endowed with significant antibacterial activities that explain at least in part its wound healing activity.

## Figures and Tables

**Table 1:** Effect of topical application of the 80% methanolic extract of the leaves of *A. polystachyus *on wound contraction of excision wound model in mice.

	**Days**	**SO**	**NF**	**5**%** CEO**	** 10**%** CEO**
**Wound area (mm** ^**2**^ **), after wounding days**	0	301.21 ± 6.14	301.21 ± 6.14	298.70 ± 6.84	301.21 ± 6.14
	2	278.81 ± 9.13	264.55 ± 12.29	273.71 ± 6.12	264.02 ± 6.12
	4	240.61 ± 6.14	210.38 ± 12.7	222.81 ± 8.16	209.60 ± 5.46
	6	214.18 ± 8.62	155.96 ± 15.43_ _^a2^	165.51 ± 7.55_ _^a1^	123.63 ± 9.97_ _^a3c1^
	8	169.56 ± 9.56	91.32 ± 11.85_ _^a3^	98.78 ± 8.57_ _^a3^	64.89 ± 8.17_ _^a3^
	10	130.71±12.31	48.80 ± 4.65_ _^a3^	52.99 ± 5.05_ _^a3^	24.08 ± 5.39_ _^a3c1^
	12	94.07 ± 12.43	23.81 ± 4.76_ _^a3^	23.68 ± 47.09_ _^a3^	3.40 ± 1.29_ _^a3^
	14	50.89 ± 10.02	4.97 ± 1.99_ _^a3^	7.07 ± 3.18_ _^a3^	0.00 ± 0.00_ _^a3^
	16	31.01± 5.38	0.00 ± 0.00_ _^a3^	1.70 ± 1.18_ _^a3^	0.00 ± 0.00_ _^a3^

SO, simple ointment base; CEO, crude extract ointment; NF, nitrofurazone; *n* = 6 animals in each group. Values are expressed as mean ± SEM (*n = *6), one-way ANOVA.  ^a^Against control and  ^c^against 5% (w/w) hydroalcoholic extract;  ^1^*P* < 0.05,  ^2^*P* < 0.01, and  ^3^*P* < 0.001.

**Table 2:** Effect of topical application of the 80% methanolic crude extract ointment of the leaves of *A. polystachyus* D. on period of epithelialization (number of days).

Treatment groups	Period of epithelialization (days) Mean ± SEM	% decrease in epithelialization periods
Simple ointment base	20.83 ± 1.01	-
Nitrofurazone (0.2 % w/w)	15.33 ± 0.42_ _^a3^	26.4
5% crude extract ointment (w/w)	15.83 ± 0.65_ _^a3^	24
10% crude extract ointment ( w/w)	13.17 ± 0.40_ _^a3c1^	36.8

Note: values are expressed as mean ± SEM (*n* = 6), one-way ANOVA. ^a^Compared to negative control (simple ointment); ^c^compared to 5% ointment; ^1^*P* < 0.05, ^2^*P* < 0.01, and ^3^*P* < 0.001, when compared to control group, one-way ANOVA.

**Table 3:** Effect of topical application of 80% methanolic crude extract ointment of the leaves of *A*.* polystachyus on* wound contraction of infected wound model in mice.

	**Days**	**SO**	**NF**	**5**%** CEO**	** 10**%** CEO**
**Wound area (mm** ^**2**^ **), after wounding days**	0	298.20 ± 6.84	301.21 ± 6.15	298.69 ± 6.84	301.21± 6.15
	2	278.81± 9.13	259.45 ± 8.73	268.87 ± 6.50	259.19 ± 4.84
	4	254.87 ± 10.32	205.81 ± 10.09_ _^a2^	213.9 ± 5.79_ _^a1^	201.22 ± 6.49_ _^a2^
	6	214.44 ± 11.01	144.31± 11.38_ _^a2^	150.86 ± 8.81_ _^a2^	126.91± 9.81_ _^a3^
	8	186.57 ± 17.40	85.04 ± 8.72_ _^a3^	96.03 ± 9.23_ _^a3^	67.38 ± 8.47_ _^a3^
	10	156.22 ± 17.46	43.70 ± 6.69_ _^a3^	56.00 ± 8.15_ _^a3^	27.74 ± 6.98_ _^a3^
	12	120.24 ± 19.51	19.63 ± 5.73_ _^a3^	27.09 ± 8.02_ _^a3^	7.07 ± 3.18_ _^a3^
	14	107.02 ± 18.87	5.63 ± 3.14_ _^a3^	11.12 ± 5.52_ _^a3^	1.70 ± 1.19_ _^a3^
	16	85.31 ± 18.59	0.66 ± 0.51_ _^a3^	2.49 ± 3.56_ _^a3^	0.00 ± 0.00_ _^a3^
	18	68.04 ± 16.99	0.00 ± 0.00_ _^a3^	0.00 ± 0.00_ _^a3^	0.00 ± 0.00_ _^a3^

Note: SO, simple ointment base; CEO, crude extract ointment; NF, nitrofurazone; *n* = 6 animals in each group. Values are expressed as mean ± SEM (*n = *6), one-way ANOVA.  ^a^Against negative control,  ^1^*P *< 0.05,  ^2^*P* < 0.01, and ^3^*P* < 0.001.

**Table 4:** Effect of topical application of the 80% methanolic extract ointment of *A. polystachyus* on the period of epithelialization of infected wound model in mice.

Treatment groups	Period of epithelialization (days)
Simple ointment base	FR
Nitrofurazone (0.2 % w/w)	16.17 ± 1.83_ _^a3^
5% crude extract ointment (w/w)	17.00 ± 0.86_ _^a3^
10% crude extract ointment (w/w)	14.17 ± 1.17_ _^a3c1^

Note: FR: failed to reepithelize. Values are expressed as mean ± SEM (*n = *6), one-way ANOVA.  ^a^Compared to negative control (simple ointment);  ^c^compared to 5% ointment;  ^1^*P *< 0.05 and  ^3^*P *< 0.001.

**Table 5:** Effect of topical application of the 80% methanolic crude extract ointment of *A. polystachyus *leaveson tensile strength of incision wound model in mice.

Treatment group	Tensile strength (g) (mean ± SEM)	% tensile strength
Simple ointment	197.00 ± 5.25	_
Nitrofurazone (0.2%)	261.33 ± 5.89_ _^a3^	31.22
5% crude extract ointment	258.50 ± 7.21_ _^a3^	32.65
10% crude extract ointment	267.50 ± 7.61_ _^a3^	35.79

Note: values are expressed as mean ± SEM (*n = *6), one-way ANOVA.  ^a^Compared to negative (simple ointment) control;  ^3^*P* < 0.001, when compared to control group, one-way ANOVA.

## Data Availability

The original data used to support the findings of this study have been deposited in the University of Gondar repository.
